# Precision Optical Metrology and Smart Sensing

**DOI:** 10.3390/s24216816

**Published:** 2024-10-23

**Authors:** Xiangchao Zhang, Jian Wang, Yajun Wang

**Affiliations:** 1 Shanghai Engineering Research Center of Ultra-Precision Optical Manufacturing, Fudan University, Shanghai 200438, China; zxchao@fudan.edu.cn; 2 State Key Lab of Intelligent Manufacturing Equipment and Technology, Huazhong University of Science and Technology, Wuhan 430074, China; 3 College of Electronics and Information Engineering, Sichuan University, Chengdu 610065, China; yjwangisu@scu.edu.cn

Optics, renowned for its non-contact measurement capabilities, versatility, and high sensitivity, is recognized as a powerful tool driving the technological evolution of precision metrology. It has revolutionized a wide range of industries, from semiconductor manufacturing and material science to healthcare and environmental monitoring. In combination with advanced data processing, communication technologies, and often artificial intelligence (AI), smart sensing technology has emerged as a transformative technology in fields such as industrial automation, transportation, and the Internet of Things. While precision metrology and smart sensing develop in parallel, they serve distinct roles: the former focuses on enhancing measurement accuracy or precision, typically in controlled environments, whilst the latter emphasizes real-time data acquisition and interpretation, often in dynamic uncontrolled environments. However, both fields are advancing rapidly, driven by breakthroughs in optoelectronics and high-performance computing, positioning them to address increasingly complex challenges in measurement and sensing.

This Special Issue on Precision Optical Metrology and Smart Sensing, initiated in 2023, aims to highlight the dawn of a new era in optical metrology. After a careful review, ten papers have been finally accepted for publication, with three contributions from Europe and the rest from China. These papers cover a range of topics, with one on the nonlinear effects of fundamental optoelectronic devices, three on fringe projection/photographic profilometry, four on interferometric/holographic profilometry, and two on AI-assisted image and point-cloud algorithms.

Paper [1], by G. Xie et al., from Shenzhen University, Shenzhen Sincevision Technology Ltd., and China Astronaut Research and Training Center, China, presents a novel method combining DBSCAN and a percentile filter to improve the accuracy of laser welding depth measurements using Optical Coherence Tomography (OCT). By treating noise as an outlier and filtering it out using density-based clustering (DBSCAN), the method enables efficient noise removal. The percentile filter then accurately extracts the welding depth from the cleaned data, achieving an average error of less than 5% compared to actual measurements. The proposed approach enhances the precision and practicality of OCT for real-time quality assurance in laser welding, particularly in high-precision manufacturing industries like power battery production.

Paper [2], by W. Sun et al., from Nanjing University of Aeronautics and Astronautics, China, introduces a novel hybrid fringe projection profilometry (FPP) and Digital Image Correlation (DIC) method for simultaneous 3D shape and deformation measurements using a single 3CCD color camera. By capturing blue fringe patterns and red fluorescent speckles in the same image, it reduces crosstalk and corrects displacement coupling. The method achieves high accuracy, with discrepancies as low as 0.7 μm for in-plane and 0.034 mm for out-of-plane displacements. The approach is validated through experiments and offers an efficient, precise solution for static measurements, with potential for further optimization for dynamic applications using advanced signal processing and faster projection techniques.

Paper [3], by G. Hu et al., from Hefei University of Technology, Anhui University of Science and Technology, and University of Science and Technology of China, China, presents a method to reduce motion artifacts in phase-shift profilometry (PSP), which is crucial for high-precision 3D shape measurements of moving objects. By leveraging feature information from images, the proposed method reduces phase errors caused by object motion, significantly improving measurement accuracy. The approach was validated through copper tube vibration experiments at a frequency of 320 Hz, demonstrating its effectiveness in dynamic scenarios where motion amplitudes are large. While offering superior results for objects with non-uniform velocities, the method shows limitations in handling complex textures and achieving sub-pixel optimization, with future work proposed to integrate machine learning for enhanced dynamic measurements.

Paper [4], by X. Ma et al., from Fudan University, China, introduces a novel Digital Holographic Microscopy (DHM) method to accurately measure high-slope micro-nano structures. To overcome the challenge of dense interference fringes caused by steep surface angles, the authors propose tilting the reference wavefronts to sparsify the fringes. A data fusion strategy, including region extraction and tilt correction, allows for precise surface topography reconstruction with nanometer-scale vertical resolution. The technique offers a flexible and low-cost solution for measuring complex, high-slope structures without the need for advanced modulation elements, making it suitable for applications in fields such as biology, materials science, and microelectronics.

Paper [5], by J. Wang et al., from Huazhong University of Science and Technology, China, and University of Huddersfield, IMA Ltd., and University of Nottingham, UK, presents a novel algorithm, LAST (Locally Accelerated Stitching T-spline), for fast, large-scale fitting of freeform point clouds. The method divides large-scale point clouds into smaller patches, fits each patch to a T-spline using a locally accelerated scheme, and then stitches the patches together with a local optimization approach. This strategy significantly improves computational efficiency, achieving a three-to-eightfold speed increase compared to global and local fitting algorithms, and a two-to-fourfold improvement over the latest split-connect algorithm. The proposed method reduces control point usage by 20%, offering a balance between accuracy and efficiency, with potential applications in advanced T-spline models and optical freeform surfaces.

Paper [6], by V. Moya-Zamanillo and J. Rosado, from Universidad Complutense de Madrid, Spain, presents a comprehensive study of the nonlinear response of Silicon Photomultipliers (SiPMs) using Monte Carlo (MC) simulations. The MC simulations were validated with experimental data for two SiPMs, and the study identifies key factors influencing nonlinearity, such as photon rate, pixel recovery time, light pulse shape, and readout circuit impedance. While correlated noise minimally impacts nonlinearity, it significantly affects the SiPM output current shape. The authors propose two phenomenological fitting models to describe the nonlinear response for various light pulse types, achieving an accuracy level of a few percent across a broad range of conditions, improving the understanding and modeling of SiPM behavior.

Paper [7], by K. Ma et al. from North China University of Water Resources and Electric Power, China, introduces a high-accuracy close-range photogrammetric technique for measuring the thermal deformation of a satellite antenna’s surface. The study develops a comprehensive measurement test scheme and demonstrates that the method achieves an error of less than 0.04 mm, meeting the required accuracy. Thermal deformation was found to increase as temperatures dropped, with the highest surface shape deformation occurring at −60 °C, but remaining within design specifications (RMSE ≤ 1 mm). The findings provide important insights into the deformation behavior of satellite antennas in extreme temperatures, offering practical guidance for future antenna design and performance assessments in orbit.

Paper [8], by D. Litwin et al., from Łukasiewicz Research Network and Central Office of Measures, Poland, introduces a novel multiwavelength interferometer in reflected-light mode, utilizing two Wollaston prisms to measure the step height of standards. The innovation lies in continuously measuring fringe periods and phases relative to the zero-order fringe, eliminating the need for traditional fringe coincidence searches. The study presents both theoretical and experimental validation, showing that the Equal Thickness Method (ETM) significantly outperforms classical approaches in terms of accuracy. This compact interferometer, featuring common-path wave propagation and no moving parts, is highly adaptable for various materials and environments, making it suitable for industrial applications and ensuring stable, precise measurements even under varying conditions.

Paper [9], by J. Wei et al. from Shanghai Institute of Measurement and Testing Technology and Shanghai University, China, introduces a Standard-Deviation-Based Adaptive Median Filter (SAMF) to eliminate batwing effects in step microstructure measurements using digital holography. The proposed filter dynamically adjusts its window size based on the position within the batwing effect range, determined by the standard deviation, while maintaining smaller windows outside this range to prevent distortion. Experiments on the Standard Resolution Target USAF 1951 and step height targets show that the method effectively removes batwing artifacts while preserving the integrity of the remaining profile, significantly improving measurement accuracy in digital holography for precision metrology and high-resolution surface characterization.

Paper [10], by S. Kaderuppan et al., from Newcastle University, Northumbria University, UK, and Singapore Institute of Technology, Singapore, introduces Θ-Net, a novel deep neural network (DNN) architecture designed to enhance the resolution of phase-modulated optical micrographs (e.g., PCM and DIC images) without requiring additional physical modifications or fluorescence techniques. By concatenating three O-Net architectures, Θ-Net achieves improved image resolution, outperforming popular models like ANNA-PALM, BSRGAN, and 3D RCAN in resolving fine details under poor signal-to-noise conditions. Cross-domain learning further enhances its ability to generate highly accurate images that approximate ground truth images. The proposed model has broad applications, from biomedical imaging to precision engineering and optical metrology, with potential for future use in LiDAR and remote sensing.

Together, these papers cover fundamental device physics, high-precision measurement techniques, and advanced algorithms, all of which are driving the technological revolution in precision optical metrology and smart sensing. Specifically, studies on nonlinear effects, representing cutting-edge advancements in device physics, contribute to the performance of precision measurement and smart sensing. Fringe projection and photographic profilometry are widely used for 3D shape measurement at the macro scale, while interferometric and holographic profilometry are essential for high-precision 3D topography measurements at the microscopic scale. Additionally, AI-assisted image and point-cloud algorithms reflect the shift toward more intelligent and automated systems, aligning with the broader evolution of smart sensing technologies. Therefore, we believe this collection provides a representative snapshot of the current advancements in these fields.

We would like to extend our heartfelt thanks to the authors of the ten papers for their valuable contributions, showcasing cutting-edge research in precision optical metrology and smart sensing. We are also deeply grateful to the reviewers for their thorough comments and constructive suggestions, which greatly enhanced the quality of this Special Issue. Lastly, our sincere appreciation goes to the Sensors Journal Office of MDPI for their policy guidance and financial support, without which this Special Issue would not have been possible.
